# Complete plastid genome of *Primula calliantha* Franch. (Primulaceae): an alpine ornamental plant endemic to Hengduan Mountain, China

**DOI:** 10.1080/23802359.2021.1963868

**Published:** 2021-08-13

**Authors:** Xuedan Yang, Yuan Huang, Zhonghu Li, Jiahui Chen

**Affiliations:** a School of Life Sciences, Northwest University, Xi'an, P. R. China; b CAS Key Laboratory for Plant Diversity and Biogeography of East Asia, Kunming Institute of Botany, Chinese Academy of Sciences, Kunming, P. R. China; c School of Life Sciences, Yunnan Normal University, Kunming, P. R. China

**Keywords:** Endemic, ornamental plant, *Primula*, plastid complete genome

## Abstract

*Primula calliantha* Franch. is an alpine species with an ornamental value that is endemic to the Hengduan Mountains of southwest China. Here, we sequenced and assembled its plastid complete genome, which is a circular molecule of 151,954 bp and contains a large single-copy (83,820 bp) and a small single-copy (17,814 bp) region, separated by a pair of inverted repeats (25,160 bp). There are 131 genes, 86 protein-coding, 37 tRNAs, and 8 rRNAs in the plastome, of which 114 genes, 80 CDSs, 30 tRNAs, and 4 rRNAs are unique, respectively. The *P. calliantha* plastid genome shows a high level of synteny with its close relatives, *P. chionantha*, *P. purdomii*, and *P. woodwardii*. Phylogenetic analysis based on 60 complete chloroplast genomes of Primulaceae confirmed its delimitation in *Primula*.

*Primula calliantha* is a species of *Primula* classified in section *Crystallophlomis* Rupr. of the Primulaceae (Hu and Kelso 1996). It is endemic to the Hengduan Mountains biodiversity hotspot of southwest China, growing on pastures and meadows among *Rhododendron* or on rocks of steep humus-clad slopes at elevations 3700–4500 m (Hu and Kelso 1996). This alpine endemic species is specifically distributed in the Cang-Shan Mountain of Yunnan Dali, but extends to the Shangri-La country and southeast Xizhang (Richards 2003). The flower of *P. calliantha* has 1–4 umbels, each with three to many purplish violet corolla (Hu and Kelso 1996). This species therefore exhibits high ornamental value. To provide scientific basis for the rational utilization and conservation of *P. calliantha*, we sequenced and assembled the complete plastid genome.

Fresh leaves of *P. calliantha* were collected from wild plants in Dali County, Yunnan (N 25.67°, E 100.09°) and the corresponding voucher specimen was deposited in the Herbarium of Yunnan Normal University under the accession number of HY202013 (Kunming, China; Prof. Yonghong Zhang, daphnecn@aliyun.com). Total genomic DNA was extracted from the isolated chloroplasts using a modified CTAB method (Porebski et al. 1997). According to the criterion, we fragmented the DNA and used Illumina Hiseq X Ten sequencer to construct the genomic library for Illumina paired-end (PE) sequencing. A total of 29,141,918 filtered reads were retrieved and used to assemble the plastid genome with NOVOPlasty v4.3.1 (Dierckxsens et al. 2017). Using *P. chrysochlora* Balf. F. et Ward as the reference genome (GenBank accession No: NC_034678), the assembled plastid genome was annotated using GeSeq (Tillich et al. 2017).

The complete plastid genome of *P. calliantha* is a circular molecule with a length of 151,954 bp, with an average coverage of 430, and GC content of 37.0%, respectively. The NCBI accession number of the plastid genome is MZ054238, and the Illumina sequence data are deposited in the NCBI SRA database under accession number SRR14454273. The plastid genome of this species has a large single-copy region of 83,820 bp and a small single-copy region of 17,814 bp, which are separated by a pair of inverted repeats of 25,160 bp. In total, the plastid genome has 131 genes, 86 protein-coding (CDS), 37 tRNAs, and 8 rRNAs are annotated in the plastome, of which 114 genes, 80 CDSs, 30 tRNAs, and four rRNAs are unique, respectively. The gene content, synteny of plastid genome was similar between *P. calliantha* and its close relatives, *P. chionantha*, *P. purdomii*, and *P. woodwardii*, but slightly differs in plastome length. Moreover, compared with *P. calliantha* and *P. woodwardii*, *P. chionantha* and *P. purdomii* lack the *rp12* gene (Ren et al. 2018).

To determine the phylogenetic position of *P. calliantha* within the genus *Primula*, 57 *Primula* sequences were downloaded as well as two *Androsace* species to serve as outgroups from GenBank. The plastid sequences were aligned using MAFFT (Katoh and Standely 2013), and a maximum-likelihood (ML) tree was constructed using IQ-TREE-2 (Minh et al. 2020; Katoh and Standley 2013). The best fitted model according to the Bayesian information criterion was TVM + F+R10 using ModelFinder (Kalyaanamoorthy et al. 2017). The branch support values were tested using the ultrafast bootstrap (UFBoot) setting (Hoang et al. 2018) and SH-like approximate likelihood ratio test (SH-aLRT) (Guindon et al. 2010) with 10,000 replicates (Figure 1). The ML tree showed that the 58 *Primula* species analyzed in this study form a robust monophyletic clade, and *P. calliantha* is fully resolved in a clade with four species classified to *Primula* section *Crystallophlomis*, and is sister to *P. chionantha, P. purdomii, P. woodwardia*. In contrast, a previous barcode phylogenetic analysis (Yan et al. 2015) based on *rbcL*, *matK*, and ITS sequence data revealed that *P. calliantha* is sister to *P. boreiocalliantha*, and a clade consist of these two species is sister to *P. agleniana*. However, plastomes of *P. boreiocalliantha* and *P. agleniana* are not available, and our research cannot confirm this relationship. Topological structures shown by our phylogenetic tree are generally in agreement with previously phylogenetic studies of *Primula* with some inconsistencies (Conti et al. 2000; Mast et al. 2001; Kovtonyuk and Goncharov 2009; Yan et al. 2015; Ren et al. 2018). For example, previous study revealed that sect. *Proliferae* Pax (Ren et al. 2018) and sect. *Aleuritia* (Conti et al. 2000) are monophyletic. But our results with more or different species sampled for these sections revealed they are not monophyletic, indicating that lack of samples affect the results of the phylogenetic analysis. Molecular phylogenetic studies of *Primula* so far only sampled a small fraction of species in this genus, or used few DNA marks and therefore cannot provide sufficient resolution (Conti et al. 2000; Mast et al. 2001; Kovtonyuk and Goncharov 2009; Ren et al. 2018), it is therefore arbitrary to discuss intra-generic systems (e.g. subgenus, section) of *Primula.* Nevertheless, all these studies indicated that *Primula* is a robust monophyletic clade, but most sections of *Primula* are not monophyletic, all of the relationships revealed by molecular evidence between *Primula* subgenus, sections, and species generally do not correlate with any *Primula* systems based on morphology (Richards 1993; Mast et al. 2001).

**Figure 1.**
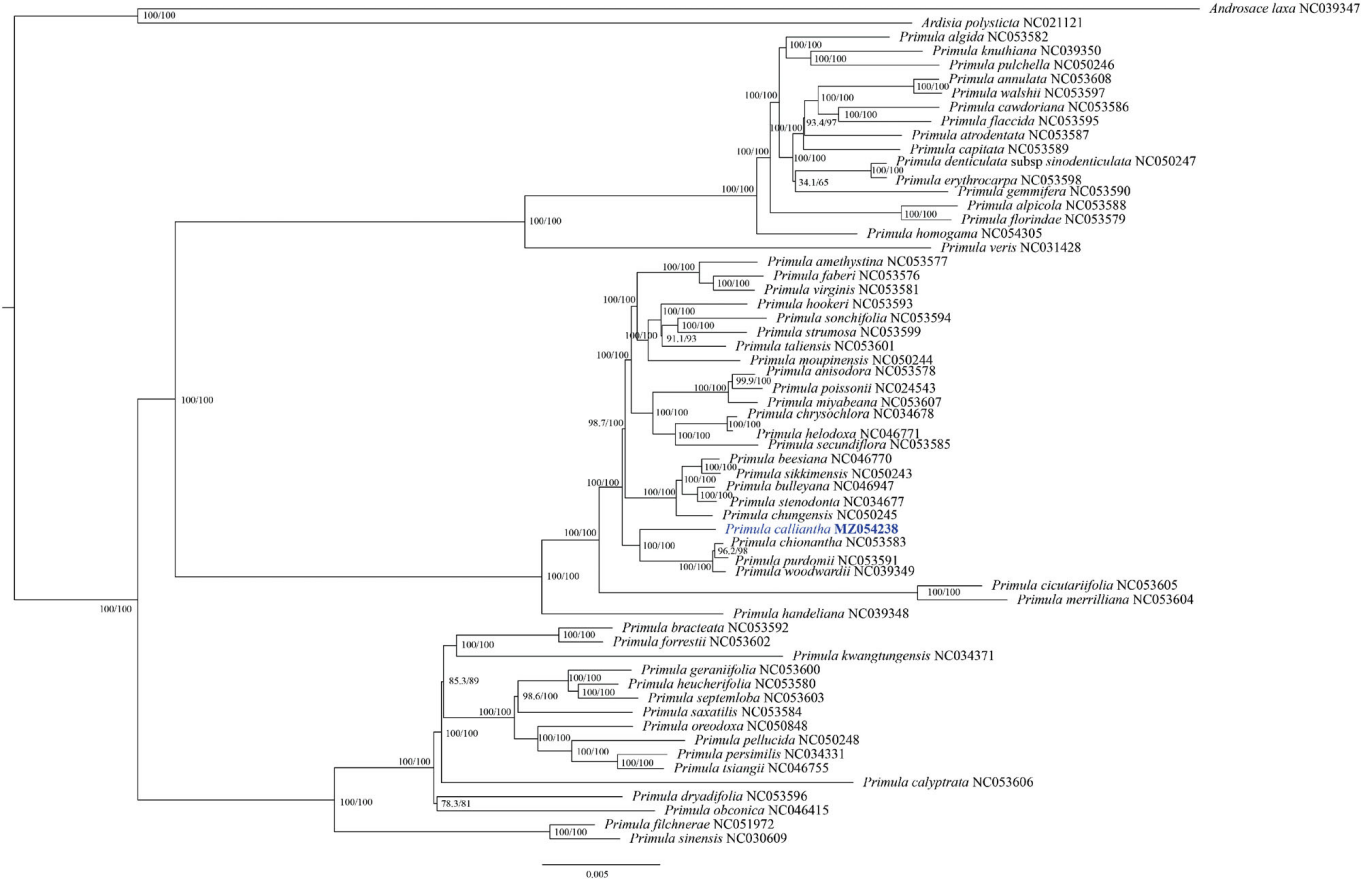
ML phylogenetic tree of *Primula calliantha* and 60 Primulaceae species based on complete plastid genomes; branch supports values are reported as SH-aLRT/UFBoot.

## Data Availability

The data that support the findings of this study are available in NCBI at https://www.ncbi.nlm.nih.gov/, reference number [MZ054238]. The associated BioProject, SRA, and Bio-Sample numbers are PRJNA727726 SRR14454273 and SAMN19030661, respectively.
